# Relationship Between Gender Differences and Clinical Outcome in Patients With the Antiphospholipid Syndrome

**DOI:** 10.3389/fimmu.2022.932181

**Published:** 2022-07-04

**Authors:** Simona Truglia, Antonella Capozzi, Silvia Mancuso, Valeria Manganelli, Luca Rapino, Gloria Riitano, Serena Recalchi, Serena Colafrancesco, Fulvia Ceccarelli, Tina Garofalo, Cristiano Alessandri, Agostina Longo, Roberta Misasi, Fabrizio Conti, Maurizio Sorice

**Affiliations:** ^1^ Reumatologia, Dipartimento di Scienze Cliniche Internistiche, Anestesiologiche Cardiovascolari, Sapienza University, Rome, Italy; ^2^ Dipartimento di Medicina Sperimentale, Sapienza University, Rome, Italy

**Keywords:** gender, antiphospholipid (Hughes) syndrome, clinical manifestations, antiphospholipid antibodies, thrombosis

## Abstract

Antiphospholipid syndrome (APS), characterized by artherial and/or venous thrombosis, pregnancy morbidity and “antiphospholipid” antibodies (aPLs), is more common in women than in men, with a female to male ratio of about 3.5:1. Only few studies have investigated the clinical differences between male and female patients with APS. Therefore, this study was aimed to analyze the differences of clinical manifestations and laboratory tests, at diagnosis, between female and male APS patients and the clinical outcome. We enrolled 191 consecutive APS patients (125 with primary APS, PAPS, and 66 with secondary APS, SAPS) with a female predominant ratio of approximately 3:1 (142 vs 49). The prevalence of PAPS was higher in males than females (p<0.001). The analysis of aPL profile revealed that high IgM anti-cardiolipin (aCL) and high-medium IgG aCL titers were more frequent in males. In thrombotic APS peripheral arterial thrombosis was more common in male than female patients (p=0.049), as well as myocardial infarction (p=0.031). Multivariate analysis to correct for cardiovascular risk factors, high titer of aPLs and triple positivity for aPLs, revealed that the odds ratio for myocardial infarction in male was 3.77. Thus, APS may be considered as a disease in which serological (IgM titer) and clinical profiles are influenced by gender.

## Introduction

Antiphospholipid syndrome (APS) is characterized by autoantibodies directed against phospholipids and/or phospholipid-binding proteins. These can be detected by the identification of anti-cardiolipin (aCL) and anti-β2-glycoprotein I (aβ2-GPI) antibodies and by the Lupus Anticoagulant (LA) coagulation test (1–4). The main clinical variants of the syndrome are two: obstetric APS, characterized by pregnancy morbidity, and vascular or thrombotic APS, characterized by the occurrence of venous and arterial thrombosis (5, 6). Other additional symptoms may include thrombocytopenia, nephropathy, cutaneous manifestations (livedo reticularis) and central nervous system symptoms, such as cognitive abnormalities and epilepsy (7, 8). In less than 1% of cases a catastrophic APS (CAPS), characterized by systemic microangiopathy is observed (9, 10). It can be present as an isolated disorder (“primary” APS, PAPS) or can appear in association with SLE or other autoimmune diseases (“secondary” APS, SAPS) (11–14). PAPS patients have a mean age at diagnosis of about 35-40 years and the disease is more common in women than in men, with a female to male ratio of about 3.5:1 (15). Despite fetal loss is a major clinical manifestation of APS, nevertheless, it is interesting to study the gender differences in patients who reported the thrombotic event only, since the data in this regard are still to be investigated (16).

So far, only few studies have investigated the clinical differences between male and female APS patients (17–20). Female APS patients showed greater central nervous system involvement with stroke/transient ischemic attack (TIA), whereas males had a prevalence of mesenteric thrombosis and Budd-Chiari syndrome or gastrointestinal complication (17). Furthermore, other arterial thrombotic events, such as myocardial infarctions and peripheral thrombosis of lower limbs, have been described more frequently in men (18). On the contrary, pulmonary embolism has been reported more frequently in women (19). It should be noted, however, that due to the rarity of the condition, these studies evaluated very small cohorts of patients. A recent paper analyzed a larger cohort of APS patients, showing a prevalence of venous thrombosis in women at a young age, while males suffered more from later-onset but more relapsing arterial events. These gender differences were related to the presence of additional risk factors, rather than to biological gender-specific issues. No gender differences were found in the anti-phospholipid antibody (aPL) profile of aPL single, double or triple positivity, evaluating the three laboratory diagnostic tests (aCL, aβ2-GPI and LA) (20).

Therefore, the aim of this study was to analyze the differences and similarities of clinical manifestations and laboratory tests, at the time of diagnosis, between female and male APS patients, also evaluating the clinical outcome.

## Patients and Methods

### Patients

This is a single center observational retrospective cohort study on adult patients with a diagnosis of APS according to the Sydney classification criteria (1).

We included all consecutive APS patients referred to the Sapienza Lupus Clinic, Rheumatology Unit of Sapienza University of Rome between the years 2008 and 2021. Data on demographic, clinical and laboratory features were registered in an electronic database.

We recorded clinical features according to Sapporo classification criteria (1), extra-criteria manifestations (livedo reticularis, thrombocytopenia, migraine, seizures, Raynaud’s phenomenon, multiple sclerosis-like syndrome, glomerular thrombosis), cardiovascular risk factors, evaluated at APS diagnosis (hypercholesterolemia, smoking, hypertension, diabetes) and laboratory tests. Patients were diagnosed to have CAPS if they presented with multiple organ involvement, simultaneously or in less than one week with thrombosis in small vessels (21). Antiphospholipid antibodies titer was defined as follow, low titer aCL/aβ2-GPI ≥20 <40 Units (GPL, MPL, UA/ml), medium titer aCL/aβ2-GPI (GPL, MPL, UA/ml) ≥40 <80 Units, high titer aCL/aβ2-GPI (GPL, MPL, UA/ml) ≥80 Units.

This study was approved by the local ethic committees and participants gave written informed consent.

### Anti-Cardiolipin and Anti-β2-Glycoprotein I antibody Assays

A QUANTA LiteTM detection kit (INOVA Diagnostic Inc., San Diego, CA, USA) assay was used to detect IgG and IgM aCL (GPL and MPL, respectively) and anti-β2-GPI antibodies (UA/ml), using the ELISA technique. ELISA was performed for all the patients’ sera according to manufacturer’s instructions; to confirm the specificity of the results, a positive control and several normal human sera were run in the same assay.

IgG and IgM aCL and aβ2-GPI antibodies were confirmed by chemiluminescence assay, using Zenit RA Immunoanalyzer (A. Menarini Diagnostics, Florence, Italy).

### Lupus Anticoagulant Test

All the patients’ plasma samples were tested for Lupus Anticoagulant, which was studied in two coagulation systems. At first, a dilute sensitized activated partial thromboplastin time and a dilute Russell’s viper venom time were performed, followed then by confirm test. Reagents and instrumentation were provided by Hemoliance Instrumentation Laboratory, Lexington, MA, USA.

### Statistical Analysis

Data are expressed as mean (standard deviation-SD-) or median (interquartile range-IQR-) according to values distribution. The χ2-test or Fisher exact test was utilized for comparison of categorical variables and Mann-Whitney U test to evaluate continuous variables. We performed the multivariate logistic regression for examining the relationship of a binary (or dichotomous) outcome (peripheral arterial thrombosis and myocardial infarction) and to investigate the risk factors associated to gender. In the multivariate analyses we included: cardiovascular risk factor, aPLs high titer, triple positivity for aPLs.

Odds ratios (ORs) and 95% confidence intervals (95% CIs) were calculated. P-Values of less than 0.05 were considered statistically significant. SPSS 27.0 statistical software package (SPSS Inc., Chicago, IL, USA) was utilized.

## Results

We enrolled 191 consecutive APS patients (125 PAPS and 66 SAPS) with a median age at onset of 38 years (IQR 17) and a female predominant ratio of approximately 3:1 (142 females and 49 males).

In the whole cohort, 109 (76.8%) females and 49 (100%) males presented thrombotic manifestations, 53 (37.3%) patients reported pregnancy morbidities and 20 (14.1%) patients mixed thrombotic and obstetrical features. The most common presenting manifestations were peripheral venous thrombosis (n= 69, 93.2%) followed by ischemic stroke (n= 31, 28.4%) in females and peripheral venous thrombosis (n= 27, 87%) followed by pulmonary embolism (n= 13, 41.9%) in males. CAPS occurred only in 2 male patients.

The demographic, clinical and laboratory features of female and male APS patients were compared as shown in **Table 1**. From the comparison, it was found that the prevalence of PAPS was higher in males than females (85.7% *vs* 58.5%, p<0.001), indeed the ratio of PAPS to SAPS in men was 6:1 and in women it was 1.4:1. Hydroxychloroquine intake was higher in female than in male APS patients (38% *vs* 20.4%, p=0.024), probably due to the fact that hydroxychloroquine is commonly used in SLE patients, unless contraindicated.

**Table 1:** Difference between female and male patients with APS.

	Female APS (n=142, 74.3%)	Male APS (n=49, 25.7%)	p-value
**Median age in years (IQR)**	37.5 (16)	38 (19.5)	0.874
**PAPS** **SAPS** SLE Others autoimmune diseases	83 (58.5) 59 (41.5) 48 (81.4) 11 (18.6)	42 (85.7) 7 (14.3) 2 (28.6) 5 (71.4)	**<0.001** **<0.001** **<0.001** **<0.001**
***Cardiovascular risk factors, n (%)***			
Cardiovascular risk factors Hypercholesterolemia Smoking Hypertension OC/HRT Diabetes	79 (55.6) 45 (57) 10 (12.7) 49 (62) 6 (7.6) 7 (8.8)	34 (68.4) 20 (58.8) 13 (38.2) 26 (76.5) - 3 (8.8)	0.128 0.245 **<0.001** **0.022** **-** 0.747
***Antiphospholipid antibodies, n (%)***			
Anti-cardiolipin antibodies IgM Low titer Medium titer High titer Anti-cardiolipin antibodies IgG Low titer Medium titer High titer Anti-β2-glycoprotein I antibodies IgM Low titer Medium titer High titer Anti-β2-glycoprotein I antibodies IgG Low titer Medium titer High titer Lupus Anticoagulant* Triple positivity*	62 (43.7) 19 (30.6) 30 (48.4) 13 (21) 109 (76.8) 13 (11.9) 51 (46.8) 45 (41.3) 51 (35.9) 19 (37.2) 16 (31.4) 16 (31.4) 82 (57.7) 13 (15.9) 21 (25.6) 48 (58.5) 62/133 (46.6) 37/133 (27.8)	29 (59.2) 6 (20.7) 10 (34.5) 13 (44.8) 33 (67.3) 4 (12.1) 6 (18.2) 23 (69.7) 21 (42.8) 4 (19.1) 8 (38.1) 9 (42.8) 30 (61.2) 1 (3.3) 9 (20) 20 (66.7) 27/47 (57.4) 17/47 (36.2)	0.061 0.529 0.546 **0.001** 0.193 0.330 **0.001** **0.047** 0.387 0.243 0.246 0.122 0.670 0.084 0.349 0.218 0.278 0.285
***Treatment after the diagnosis of APS, n (%)***			
Antiaggregant Vitamin K-antagonist New oral anticoagulant Hydroxychloroquine Immunosuppressant Antiaggregant + Vitamin K-antagonist	63 (44.4) 75 (52.8) 7 (4.9) 54 (38) 19 (13.3) 12 (8.4)	16 (32.7) 37 (75.5) 4 (8.2) 10 (20.4) 2 (4.1) 8 (16.3)	0.151 **0.005** 0.402 **0.024** 0.073 0.121

PAPS (primary antiphospholipid syndrome); SAPS (secondary antiphospholipid syndrome); SLE (systemic lupus erythematosus); HRT (hormone replacement therapy); OC (oral contraceptive).

*Lupus Anticoagulant was evaluated in a subset of patients not under anticoagulant therapy. Values in bold indicate a significant value.

The aPL profile was evaluated and the results showed that high IgM aCL and high-medium IgG aCL titers were more frequent in males (**Table 1**).

Considering that in the literature low aPL levels were frequently observed in patients with obstetric APS (OAPS) compared to female patients with thrombotic manifestations (22, 23), we considered it useful to analyze our results also by evaluating and taking into account the titer of aPL of our cohort. Therefore, we first found no difference in aPL titer between females with thrombotic APS and OAPS (**Supplementary Table S1**). Second, we repeated the analysis in the thrombotic APS cohort, excluding OAPS patients. In the remaining group (109 females plus 49 males) we still noted that male patients presented more often high titers of IgM aCL (26.5% *vs* 9.2%, p=0.004) and medium titers of IgG aCL (12.2% *vs* 33%, p=0.006) (**Table 2**).

**Table 2:** Difference between female and male patients with thrombotic APS.

Thrombotic APS	Female (n=109)	Male (n=49)	p-value
**Median age in years (IQR)** **PAPS** **SAPS**	41 (18) 61 (56) 48 (44)	38 (19.5) 42 (85.7) 7 (14.3)	0.561 0.085 0.143
***Criteria manifestations, n (%)***			
Thrombotic manifestations			
Arterial thrombosis Peripheral thrombosis Ischemic stroke Transient ischemic attack Myocardial infarction Venous thrombosis Peripheral thrombosis Pulmonary embolism Cerebral venous thrombosis Recurrent thrombosis	45 (41.3) 10 (9.2) 31 (28.4) 4 (3.7) 6 (5.5) 73 (67) 68 (62.4) 17 (15.6) 2 (1.8) 32 (29.4)	26 (53.1) 10 (20.4) 13 (26.5) 3 (6.1) 8 (16.3) 31 (63.3) 27 (55.1) 13 (26.5) 2 (0) 10 (20.4)	0.169 **0.049** 0.804 0.375 **0.031** 0.650 0.387 0.105 0.674 0.239
***Extra-criteria manifestations, n (%)***			
Extra-criteria manifestations Livedo reticularis Thrombocytopenia Migraine Seizures Raynaud’s phenomenon Multiple Sclerosis-like syndrome Glomerular thrombosis	43 (51.8) 12 (11) 21 (19.3) 14 (12.8) 12 (11) 14 (12.8) 5 (4.6) 2 (1.8)	19 (45.2) 4 (8.2) 11 (22.4) 3 (6.1) 7 (14.3) 3 (6.1) 0 0	0.488 0.583 0.645 0.207 0.558 0.207 0.152 0.475
***Cardiovascular risk factors, n (%)***			
Cardiovascular risk factors Hypercholesterolemia Smoking Hypertension Diabetes	68 (62.4) 41 (37.6) 10 (9.2) 41 (37.6) 6 (5.5)	34 (69.4) 20 (40.8) 13 (26.5) 26 (53.1) 3 (6.1)	0.395 0.702 **0.004** 0.069 0.568
***Antiphospholipid antibodies, n (%)***			
Anti-cardiolipin antibodies IgM Low titer Medium titer High titer Anti-cardiolipin antibodies IgG Low titer Medium titer High titer Anti-β2-glycoprotein I antibodies IgM Low titer Medium titer High titer Anti-β2-glycoprotein I antibodies IgG Low titer Medium titer High titer Lupus Anticoagulant* Triple positivity*	45 (41.3) 14 (12.8) 21 (19.3) 10 (9.2) 88 (80.7) 12 (11) 36 (33) 40 (36.7) 40 (36.7) 17 (15.6) 11 (10.1) 12 (11) 49 (59) 9 (10.8) 10 (12) 30 (36.1) 43/100 (43) 26/100 (26)	29 (59.2) 6 (12.2) 10 (20.4) 13 (26.5) 33 (67.3) 4 (8.2) 6 (12.2) 23 (46.9) 21 (42.9) 4 (8.2) 8 (16.3) 9 (18.4) 26 (61.9) 1 (2.4) 7 (16.7) 18 (42.9) 27/47 (57.4) 17/47 (36.2)	**0.037** 0.917 0.867 **0.004** 0.066 0.583 **0.006** 0.224 0.462 0.203 0.265 0.208 0.757 0.162 0.477 0.466 0.102 0.206

PAPS (primary antiphospholipid syndrome); SAPS (secondary antiphospholipid syndrome); SLE (systemic lupus erythematosus); HRT (hormone replacement therapy); OC (oral contraceptive).

*Lupus Anticoagulant was evaluated in a subset of patients not under anticoagulant therapy. Values in bold indicate a significant value.

We also compared male and female patients with thrombotic APS for the clinical and laboratory features, the results showed that peripheral arterial thrombosis was more common in male than in female patients (20.4% *vs* 9.2%, p=0.049), as well as myocardial infarction (16.3% *vs* 5.5%, p=0.031). Regarding cardiovascular risk factors, tobacco use (26.5% *vs* 9.2%, p=0.004) was more frequent in males than in females (**Table 2**). Therefore, we performed a multivariate analysis to correct for cardiovascular risk factors, high titer of aPLs and triple positivity for aPLs, revealing that the OR for myocardial infarction in male was 3.77 (IC 95% 1.09-13.08, p=0.036).

In the subgroup analysis of thrombotic patients with PAPS, male patients still showed a greater prevalence of aCL IgM at high titer than females, while aCL IgG at medium titer occurred more often in females (32.5% *vs* 7.1%, p=0.002), tobacco use was more frequent in males than in females (**Table 3**).

**Table 3:** Clinical and laboratory characteristics of PAPS patients with thrombotic events.

	Female (n=61, 59.2%)	Male (n=42, 40.8%)	p-value
**Median age in years (IQR)**	44 (21)	40.5 (18.5)	0.319
***Criteria manifestations, n (%)***			
**Thrombotic manifestations**			
Arterial thrombosis Peripheral thrombosis Ischemic stroke Transient ischemic attack Myocardial infarction Venous thrombosis Peripheral thrombosis Pulmonary embolism Cerebral venous thrombosis Recurrent thrombosis	28 (45.9) 7 (11.4) 18 (29.5) 1 (1.6) 4 (13.1) 39 (63.9) 35 (57.3) 10 (16.3) 2 (3.2) 19 (31.1)	24(57.1) 9 (21.4) 12 (28.5) 3 (7.1) 8 (9.5) 26 (61.9) 24 (57.1) 11 (26.1) 0 9 (21.4)	0.262 0.170 0.918 0.155 0.576 0.833 0.981 0.225 0.236 0.275
**Thrombosis + Pregnancy morbidity**	14 (23)	–	–
***Extra-criteria manifestations, n (%)***			
Extra-criteria manifestations Livedo reticularis Thrombocytopenia Migraine Seizures Raynaud’s phenomenon Multiple Sclerosis-like syndrome Glomerular thrombosis	33 (54) 8 (13.1) 9 (18.1) 10 (16.3) 6 (9.8) 6 (9.8) 1 (1.6) 1 (1.6)	19 (45.2) 4 (9.5) 11(21.4) 3 (7.1) 5 (11.9) 3 (7.1) 0 0	0.376 0.576 0.668 0.164 0.738 0.634 0.404 0.404
***Cardiovascular risk factors, n (%)***			
Cardiovascular risk factors Hypercholesterolemia Smoking Hypertension OC/HRT	38 (62.2) 27 (44.2) 5 (8.1) 22 (36)	30 (71.4) 18 (42.8) 12 (28.5) 23 (54.7) -	0.336 0.887 **0.006** 0.060 **-**
Diabetes	3 (4.9)	2 (4.7)	0.971
***Antiphospholipid antibodies, n (%)***			
Anti-cardiolipin antibodies IgM Low titer Medium titer High titer Anti-cardiolipin antibodies IgG Low titer Medium titer High titer Anti-β2-glycoprotein I antibodies IgM Low titer Medium titer High titer Anti-β2-glycoprotein I antibodies IgG Low titer Medium titer High titer Lupus Anticoagulant* Triple positivity*	24 (39.3) 9 (14.8) 11 (18) 4 (6.6) 48 (78.7) 5 (8.2) 18 (29.5) 25 (41) 24 (39.3) 13 (21.3) 6 (9.8) 5 (8.2) 37 (60.7) 6 (9.8) 6 (9.8) 25 (41) 26/57 (45.6) 16/57 (28.1)	28 (66.7) 6 (14.3) 10 (23.8) 12 (28.6) 28 (66.7) 4 (9.5) 3 (7.1) 21 (50) 20 (47.6) 4 (9.5) 8 (19) 8 (19) 26 (61.9) 1 (2.4) 7 (16.7) 18 (42.9) 24/40 (60) 16/40 (40)	**0.006** 0.947 0.475 **0.002** 0.173 0.815 **0.006** 0.366 0.404 0.113 0.180 0.103 0.898 0.140 0.305 0.850 0.163 0.219

HRT (hormone replacement therapy); OC (oral contraceptive).

*Lupus Anticoagulant was evaluated in a subset of patients not under anticoagulant therapy. Values in bold indicate a significant value.

## Discussion

The results of this gender-oriented study suggest that some clinical and laboratory differences in APS could be found between female and male.

Although epidemiological data on APS remain limited, and the gender ratio varies widely across studies, ranging from 10:1 to 1:1 (24), in our study we found a female predominant ratio of approximately 3:1.

The causes of this predominance are certainly multifactorial, mainly linked to the X chromosome and to different genes expressed exclusively on this chromosome, such as TLR7, FOX-P3, CD40L (25) and other genes involved in the process of immune response (26). Indeed, there are multiple hypotheses about the genetic mechanisms of autoimmune diseases, such as X monosomy until inactivation of X chromosome that could explain gender difference (27). Moreover, X chromosome implication is indicated in various Klinefelter’s studies where the patients have an increase of autoimmune disease (28, 29). A further contribution is provided by the influence of sex hormones and the occurrence of pregnancy that could apport immunological change, such as the shift from Th1 response to a type Th2, which could be related to the increase of progesterone level. In addition, estrogens, which bind specific receptors on lymphocytes, can affect both innate and adaptive immune system (30).

However, starting from these premises and to the best of our knowledge, this is the first gender-oriented study that included PAPS and SAPS patients and that evaluated the differences on aPL titers.

It is known that male patients are mainly affected by PAPS, in fact, in a Korean epidemiological study the ratio of PAPS to SAPS in men was about 2:1, but in females, it was 1:1.2 (31). In our cohort, PAPS was more prevalent in male patients than in females, indeed the ratio of PAPS to SAPS in men was 6:1, while in females it was 1.4:1.

Regarding the differences in clinical manifestations, two small studies are available. A Mexican study found that stroke/TIA was more prevalent in females than in males (12/28 *vs* 3/30, p=0.03) and gastrointestinal complications in male patients (7/30 *vs* 1/38, p=0.009) (17), otherwise a Brazilian study reported differences in the prevalence of pulmonary embolism which was higher in female than male patients (13/38 *vs* 0/11, p=0.024) (19).

Recently, Moschetti and colleagues reported data on a large multicenter cohort of 433 (68% females, 32% males) PAPS patients (20), demonstrating that peripheral arterial thrombosis and myocardial infarction were more frequent among males. We confirmed and extended these findings, since we observed that males had an increased risk of clinical manifestations, mainly myocardial infarction (OR=3.77) even when we corrected for cardiovascular risk factors. Moreover, serological analysis revealed higher titer of aPLs and the presence of triple positivity for aPLs in males.

Regarding the differences in aPL profile, de Carvalho and colleagues found that more females than males tested positive for IgM aCL antibodies (76.3% *vs* 36.4%, p = 0.025) (19).

Furthermore, we found no differences in the aPL profile, in agreement with the data from Moschetti and colleagues, but, evaluating the differences in aPL titers, we observed a higher prevalence of high and medium-high titers, respectively of IgM and IgG aCL in males.

In light of the literature data on the low titer aPLs and pregnancy morbidity (22, 23), we hypothesized that differences in aPL titers between female and male could be influenced by the peculiarity of OAPS. Therefore, we conducted a sub-analysis excluding OAPS patients and we again observed that male patients more often had high IgM aCL titers and medium IgG aCL titers.

Moreover, considering the gender differences in the prevalence of PAPS and SAPS, we performed a subgroup analysis to evaluate the clinical and laboratory differences, not only in the total cohort, but also in the PAPS subgroup. In the PAPS subgroup we confirmed the results showed in all cohort regarding the cardiovascular risk factors, as regards the aPL profile we observed that males had high titers of IgM aCL more frequently than females.

In conclusion, APS may be considered as a disease in which serological (i.e. IgM titer) and clinical profiles are influenced by gender. Data from this study lead us to speculate that the differences in clinical manifestations and laboratory tests, at diagnosis, between female and male patients with APS may represent prognostic and clinical outcome factors. However, further studies on the follow-up of these patients will clarify this aspect.

## Data Availability Statement

The raw data supporting the conclusions of this article will be made available by the authors, without undue reservation.

## Ethics Statement

The studies involving human participants were reviewed and approved by Ethics committee, Sapienza University of Rome - Policlinico Umberto I. The patients/participants provided their written informed consent to participate in this study.

## Author Contributions

MS, FaC, AL, and CA designed the study; ST, SM, LR, and FuC enrolled the patients; AC, SR, and GR performed the laboratory test. ST, AC, VM, and SC analyzed the data. MS, ST, RM, and TG wrote the draft manuscript. All authors read and approved the final version of the manuscript.

## Conflict of Interest

The authors declare that the research was conducted in the absence of any commercial or financial relationships that could be construed as a potential conflict of interest.

## Publisher’s Note

All claims expressed in this article are solely those of the authors and do not necessarily represent those of their affiliated organizations, or those of the publisher, the editors and the reviewers. Any product that may be evaluated in this article, or claim that may be made by its manufacturer, is not guaranteed or endorsed by the publisher.

## Abbreviations

APS, anti-phospholipid syndrome; PAPS, primary anti-phospholipid syndrome; SAPS, secondary anti-phospholipid syndrome; OAPS, obstetric anti-phospholipid syndrome; catastrophic anti-phospholipid syndrome; aPL, anti-phospholipid antibodies; aCL, anti-cardiolipin; aβ2-GPI, anti-β2-glycoprotein I; LA, lupus anticoagulant; TIA, transient ischemic attack; ELISA, enzyme-linked immunosorbent assay; SLE, systemic lupus erythematosus.
