# Supplementary material for: Relationship Between Gender Differences and Clinical Outcome in Patients With the Antiphospholipid Syndrome
Source: Front Immunol. 2022 Jul 4;13:932181. doi: 10.3389/fimmu.2022.932181 (PMC9289158; doi:10.3389/fimmu.2022.932181)
Supplement: Supplementary file 1 [file Table_1.docx]

**Table S1. Difference between the low titer of antiphospholipid antibodies in females with thrombotic APS\obstetric APS.**

| ***Antiphospholipid antibodies*** | **OAPS**  **(n=33)** | **thrombotic APS (n=109)** | **p-value** |
| --- | --- | --- | --- |
| Low titer anti-cardiolipin antibodies IgM  Low titer anti-cardiolipin antibodies IgG  Low titer anti-β2-glycoprotein I antibodies IgM  Low titer anti-β2-glycoprotein I antibodies IgG | 15%  9%  6%  12% | 13.4%  12%  13.4%  9% | p=0.839  p=0.645  p=0.148  p=0.645 |

OAPS (obstetric antiphospholipid syndrome)
